# English Bulldogs in the UK: a VetCompass study of their disorder predispositions and protections

**DOI:** 10.1186/s40575-022-00118-5

**Published:** 2022-06-15

**Authors:** Dan G. O’Neill, Alison Skipper, Rowena M. A. Packer, Caitriona Lacey, Dave C. Brodbelt, David B. Church, Camilla Pegram

**Affiliations:** 1 Pathobiology and Population Sciences, The Royal Veterinary College, North Mymms, Hawkshead Lane, Hatfield, AL9 7TA Herts UK; 2 Department of History, King’s College London, Strand, London, WC2R 2LS UK; 3 Clinical Science and Services, The Royal Veterinary College, North Mymms, Hawkshead Lane, Hatfield, AL9 7TA Herts UK

**Keywords:** VetCompass, Electronic patient record, EPR, Breed, Dog, Epidemiology, Primary-care, Veterinary, Pedigree, Purebred, English Bulldog, Health

## Abstract

**Background:**

The English Bulldog has risen sharply in popularity over the past decade but its distinctive and extreme conformation is linked to several serious health conditions. Using multivariable analysis of anonymised veterinary clinical data from the VetCompass Programme, this study compared the odds of common disorders between English Bulldogs and all remaining dogs in the UK during 2016.

**Results:**

From 905,544 dogs under veterinary care during 2016, the analysis included a random sample of 2,662 English Bulldogs and 22,039 dogs that are not English Bulldogs. English Bulldogs had 2.04 times the odds of diagnosis with ≥ 1 disorder than dogs that are not English Bulldogs (95% confidence interval [CI] 1.85 to 2.25). At a specific-level of diagnostic precision, English Bulldogs had increased odds of 24/43 (55.8%) disorders. These included: skin fold dermatitis (odds ratio [OR] 38.12; 95% CI 26.86 to 54.10), prolapsed nictitating membrane gland (OR 26.79; 95% CI 18.61 to 38.58) and mandibular prognathism (OR 24.32; 95% CI 13.59 to 43.53). Conversely, English Bulldogs had significantly reduced odds of 6/43 (14.0%) disorders. These included: retained deciduous tooth (OR 0.02; 95% CI 0.01 to 0.17), lipoma (OR 0.06; 95% CI 0.01 to 0.40) and periodontal disease (OR 0.23; 95% CI 0.18 to 0.30). At a grouped-level of diagnostic precision, English Bulldogs had significantly increased odds of 17/34 (50.0%) disorders. These included: congenital disorder (OR 7.55; 95% CI 5.29 to 10.76), tail disorder (OR 6.01; 95% CI 3.91 to 9.24) and lower respiratory tract disorder (OR 5.50; 95% CI 4.11 to 7.35). Conversely, English Bulldogs had significantly reduced odds of 3/34 (8.8%) disorders. These were: dental disorder (OR 0.25; 95% CI 0.20 to 0.31), spinal cord disorder (OR 0.31; 95% CI 0.14 to 0.71) and appetite disorder (OR 0.43; 95% CI 0.20 to 0.91).

**Conclusions:**

These results suggest that the health of English Bulldogs is substantially lower than dogs that are not English Bulldogs and that many predispositions in the breed are driven by the extreme conformation of these dogs. Consequently, immediate redefinition of the breed towards a moderate conformation is strongly advocated to avoid the UK joining the growing list of countries where breeding of English Bulldogs is banned.

## Plain English summary

The English Bulldog has risen sharply in popularity over the past decade in the UK. However, its distinctive and extreme conformation has raised concerns because many of these physical features render the breed prone to serious health conditions. There are increasing international efforts to either reform the breed towards a more moderate conformation or, in the absence of such change, to ban the breeding of these dogs on welfare grounds.

This VetCompass study explored random samples of anonymised veterinary clinical records from 2,662 English Bulldogs and 22,039 dogs that are not English Bulldogs from an overall population of 905,544 dogs under veterinary care during 2016 in the UK to identify all health disorders recorded for each dog during 2016. The most common disorders in each group were identified and the risks for the most common disorders overall was compared between the English Bulldogs and dogs that are not English Bulldogs.

English Bulldogs were younger (2.65 years vs 4.42 years) and heavier (25.55kg vs 13.54kg) than dogs that are not English Bulldogs. English Bulldogs had 2.04 times the risk of diagnosis with at least one disorder during 2016 compared with dogs that are not English Bulldogs. English Bulldogs had increased risk for 24/43 (55.8%) disorders compared to dogs that are not English Bulldogs. The disorders with the highest risk in English Bulldogs included skin fold dermatitis (× 38.12), prolapsed nictitating membrane gland [cherry eye] (× 26.79), protruding lower jaw (× 24.32), brachycephalic obstructive airway syndrome [BOAS] (× 19.20) and interdigital cyst (× 12.96). Conversely, English Bulldogs had significantly reduced risk of 6/43 (14.0%) disorders compared to dogs that are not English Bulldogs. These included: retained deciduous [baby] tooth (× 0.02), lipoma [fatty mass] (× 0.06), dental disease (× 0.23), itchy skin (× 0.25) and flea infestation (× 0.40).

The results of this study suggest that the overall health of the English Bulldog is much lower than dogs that are not English Bulldogs. Many of the disorder predispositions reported in this study are intrinsically related to the extreme conformation of English Bulldogs. Redefining the body-shape of the breed away from its current extreme conformation and towards a moderate conformation is advocated for urgent action to avoid the UK joining the growing list of countries where breeding of English Bulldogs is banned.

## Background

The (English) Bulldog was originally a muscular and athletic animal bred and used to attack bulls for sport, broadly similar in conformation to the modern Staffordshire Bull Terrier [1]. After this activity was banned in 1835, the Bulldog breed became associated with the Victorian underworld, but was repurposed as a show dog at the end of the nineteenth century. Dog showing was a fashionable pastime at that time and was largely regulated by The Kennel Club from 1873 onwards [2]. Bulldogs, like other breeds that were ‘developed’ at that time, were selectively bred to conform with newly written ‘breed standards’, produced by the relevant breed club to describe their idealised preferred appearance [2]. Consequently, during the 1890s, the combined influences of the breed standard and show-ring fashion drove a dramatic physical transformation of the Bulldog.

This refashioned Bulldog had more exaggerated conformation than its ancestors – in particular, a shorter face with a protruding underjaw, heavier build and bowed forelegs [3]. Because this physical transformation was both dramatic and rapid, it triggered considerable controversy among breeders at that time [3]. Critics claimed that the new-style (English) Bulldog was inherently ‘delicate’ and ‘degenerate’ [4, 5]. They described many disorders then that are still reported as common problems in modern English Bulldogs, such as short lifespans, heat and exercise intolerance, dystocia, skin disease and noisy breathing [6]. While these historical accounts are inevitably subjective, lack quantitative data and are largely grounded in superseded understandings of pathology, they nevertheless demonstrate that, over a century ago, the (English) Bulldog was already showing a variety of health problems that correlate with those still reported in the breed today, and that, even then, Bulldogs were widely considered less robust than many other breeds [7]. Moreover, they also reveal that the fashion for extreme English Bulldog conformation has persisted for over a century despite widespread awareness of the linked health issues [8]. Because of the preexisting significance of the breed as a nationalistic icon, the new body shape of the (English) Bulldog was widely depicted in patriotic imagery from 1900 onwards, and became firmly established in popular culture thereafter [3, 9]. Although (English) Bulldogs have continued to change physically since then, they therefore still retain many physical attributes (such as facial skin folds or the ‘screw’ tail) that were considered desirable novelties in the late Victorian show-ring. Many of these physical attributes remain accepted and/or valued by many breed enthusiasts, and popular with the public, today, despite growing evidence of their associations with disease [6, 10–14]. Indeed, brachycephalic breeds, including the English Bulldog, are currently experiencing a surge in popularity, paradoxically despite increasing evidence and awareness of serious health problems linked to their physical appearance, such as respiratory compromise [12, 14], spinal defects [15], dystocia [11], ocular disorders [13, 16, 17] and skin disease [18].

From the start of the twentieth century, veterinary canine specialists freely acknowledged the (English) Bulldog’s predilection to conformation-related disease [3, 19]. As small animal practice gained political influence during the 1960s, leaders of the newly-formed British Small Animal Veterinary Association (BSAVA) undertook a pioneering survey of breed-related disease in pedigree dogs, which provides some limited quantitative data on Bulldog health at that time [20]. In 1962, (English) Bulldogs were the 34^th^ most numerous breed registered by the Kennel Club, comprising 0.5% of all registrations that year [21]. Yet they were disproportionately reported as affected with several breed-related conditions, comprising 5% of all reported cases of entropion, 17% of all reported cases of skin fold dermatitis (exceeded only by the then far more numerous Cocker Spaniel and Pekingese) and 62.5% of all reported cases of ‘prolonged soft palate’ (then a newly-described condition that broadly equates to a major component of brachycephalic obstructive airway syndrome [BOAS] today) [20, 22]. Despite the methodological limitations of this early survey, it provides strong evidence that, sixty years ago, English Bulldogs were disproportionately affected by several conformation-related diseases, which, like those that were already being mentioned half a century earlier, broadly mirror those still reported as common in the breed today [6].

During the last decade, following the recommendations of the Bateson report [23], there have been increasing efforts to obtain accurate quantitative data on the frequency of breed-related disease in purebred/pedigree dogs. Using anonymised clinical data from primary care veterinary clinics in the UK, the VetCompass Programme [24] has previously reported high levels of skin and ear disease, ophthalmological disorders and respiratory issues in English Bulldogs [10]. In parallel, the Kennel Club Health and Welfare Team, liaising with health-prioritising breeders within the (English) Bulldog breed community, have produced a Bulldog Breed Health and Conservation Plan (BHCP) which similarly lists BOAS, eye problems and skin conditions as the ‘top health and welfare concerns’ within the breed for 2019 [25], echoing both the conclusions from previous VetCompass analyses [10] but also the breed predispositions to disease highlighted in the original 1962 BSAVA survey [20, 26]. Although previous research, historical evidence and anecdotal experience all support a general conclusion that English Bulldogs suffer extremely high levels of conformation-related disease, there remains a need to provide reliable supporting evidence to quantify this conclusion. Growing concerns about the welfare of dogs with severely brachycephalic conformation promoted the formation of the Brachycephalic Working Group in the UK in 2016, followed by the International Collaborative for Extreme Conformations in Dogs in 2019 [27, 28]. Legislative action has also been taken in countries such as the Netherlands where breeding of 12 brachycephalic dog breeds was banned in 2014 [29, 30] and Norway where breeding of English Bulldogs was banned in 2022 [31].

Using anonymised veterinary clinical data from the VetCompass Programme [24], this study aimed to report the most commonly recorded disorders in English Bulldogs and to compare the odds of common disorders between English Bulldogs and all remaining dogs under primary veterinary care in the UK during 2016 after accounting for major confounding variables. Based on prior evidence of frequent health issues in the breed [10], the study hypothesised English Bulldogs would show more predispositions than protections among common disorders overall. The study also hypothesized that the disorders with the highest levels of predisposition in English Bulldogs are closely linked to the extreme conformation that defines the English Bulldog breed [8]. These results could assist breeders, veterinary practitioners and owners with a robust evidence base on the health of the wider general population of English Bulldogs to predict, prevent and manage key health and welfare opportunities for the breed.

## Methods

The study population included all available dogs under primary veterinary care at clinics participating in the VetCompass Programme during 2016. Dogs under veterinary care were defined as those with either a) at least one electronic patient record (EPR) (free-text clinical note, treatment or bodyweight) recorded during 2016 or b) at least one EPR recorded during both 2015 and 2017. VetCompass collates anonymised EPR data from primary-care veterinary practices in the UK for epidemiological research [24]. Data fields available to VetCompass researchers include a unique animal identifier along with species, breed, date of birth, sex, neuter status, insurance status and bodyweight, and also clinical information from free-form text clinical notes, summary diagnosis terms [32] and treatment with relevant dates.

A cross-sectional study design was used to estimate and compare the one-year (2016) period prevalence of the most commonly diagnosed disorders in a random sample of English Bulldogs and a random sample of all other dogs. Power calculations estimated that 2,184 English Bulldogs and 21,832 dogs that are not English Bulldogs were needed to detect an odds ratio of ≥ 1.5 for a disorder occurring in 2% of dogs that are not English Bulldogs, with 80% power and 95% confidence and assuming a 10:1 ratio of dogs that are not English Bulldogs to English Bulldogs [33, 34]. Ethics approval was obtained from the RVC Ethics and Welfare Committee (reference number SR2018-1652).

Breed information entered by the participating practices was cleaned and mapped to a VetCompass breed list derived and extended from the VeNom Coding breed list [32]. Dogs recorded as English Bulldog were categorised as English Bulldog and dogs recorded with any other breed term were categorised as dogs that are not English Bulldogs. Neuter status was defined by the final available EPR neuter value and was combined with sex (female entire, female neutered, male entire, male neutered). Adult bodyweight was defined as the mean of all bodyweight (kg) values recorded for each dog after reaching 18 months old. Mean adult bodyweight was reported overall and broken down by sex for all breeds with adult bodyweight available for at least 100 dogs. Bodyweight was further categorised as “at or above the breed/sex mean”, “below the breed/sex mean” and “no recorded bodyweight”. Age (years) at the final study date (December 31, 2016) was categorised: < 1.0, 1.0 to < 2.0, 2.0 to < 4.0, 4.0 to < 6.0, 6.0 to < 8.0 and ≥ 8.0. Veterinary group attended was categorised as 1–5, based on the 5 practice groups involved in the study. Veterinary group describes aggregations of individual veterinary practices within consolidated larger consortia and were included in the current analysis to account for confounding effects that might have arisen from the owners' choice of individual practice to attend. The veterinary groups included in the current study were assigned a code during analysis to ensure anonymity and included practices that were distributed throughout the UK. Insurance status was categorised as insured or not insured as recorded by the final available EPR.

The list of unique animal identification numbers for all dogs under veterinary care in 2016 was randomly ordered and the clinical records of a randomly selected subset of animals were reviewed manually in detail to extract the most definitive diagnoses recorded for all disorders that existed during 2016 [34]. Elective (e.g., neutering) or prophylactic (e.g., vaccination) clinical events were not included. No distinction was made between pre-existing and incident disorder presentations. Disorders described within the clinical notes using presenting sign terms (e.g., ‘vomiting’ or 'vomiting and diarrhoea'), but without a formally recorded clinical diagnostic term, were included using the first sign listed (e.g., vomiting). The extracted diagnosis terms were mapped to a dual hierarchy of diagnostic precision for analysis: specific-level precision and grouped-level precision as previously described [34]. Briefly, specific-level precision terms described the original extracted terms at the maximal diagnostic precision recorded within the clinical notes (e.g., inflammatory bowel disease would remain as inflammatory bowel disease). Grouped-level precision terms mapped the original diagnosis terms to a general level of diagnostic precision (e.g., inflammatory bowel disease would map to gastro-intestinal). Following data checking for internal validity and cleaning in Excel (Microsoft Office Excel 2013, Microsoft Corp.), analyses were conducted using SPSS version 24.0 (IBM Corp). The sex-neuter status, age, adult bodyweight and insurance status for English Bulldogs and dogs that are not English Bulldogs under veterinary care during 2016 were described.

One-year period prevalence values were reported separately for English Bulldogs and dogs that are not English Bulldogs to describe the probability of diagnosis at least once during 2016. The final combined list of common disorders included the 30 most common disorders in English Bulldogs and the 30 most common disorders in dogs that are not English Bulldogs. Categorical data were summarized with number (percent) and continuous variables were summarised using median, interquartile range (IQR) and range. Mann–Whitney U test, chi-square test and Fisher’s exact test were used as appropriate for comparison of demographic data between cases and non-cases [35]. Multivariable modelling using binary logistic regression was used to report the odds of each disorder in English Bulldogs compared with dogs that are not English Bulldogs. A separate model was created for each specific-level and grouped-level disorder. Information theory was applied to generate a list of confounding variables that was consistently included alongside the breed variable in each model [36, 37]. Confounding describes the mixing together of the effects from two or more variables on an outcome such as disorder occurrence [38]. Breed was an a priori variable of interest and was therefore included in all models. Given prior evidence of differences between English Bulldogs and dogs that are not English Bulldogs in other variables that have also been shown to be associated with disorder risk [10, 39], each disorder model also included age (years), sex-neuter status, at/above or below mean bodyweight, insurance status and veterinary group to account for confounding effects Model fit was assessed with the Hosmer–Lemeshow Test [40]. Statistical significance was set at the 5% level.

## Results

The study population of 905,544 dogs under veterinary care during 2016 in the UK included 8,410 English Bulldogs (0.93%) and 897,134 dogs that are not English Bulldogs (99.07%). Random samples of 2,662/8,410 (31.65%) English Bulldogs and 22,039/897,134 (2.46%) dogs that are not English Bulldogs were included in the analysis. Data completeness were breed 99.7%, age 98.8%, sex-neuter status 99.7%, insurance status 100.0% and bodyweight 65.5%.

Descriptive results were reported on 2,662 English Bulldogs and 22,039 dogs that are not English Bulldogs (Table 1). The median age of English Bulldogs (2.65 years, IQR 1.30 – 5.10, range 0.10 – 19.54) was younger than for dogs that are not English Bulldogs (4.42 years, IQR 1.88 – 8.10, range 0.01 – 20.46) (*p* < 0.001). The median adult bodyweight of English Bulldogs (25.55 kg, IQR 22.83 – 28.48, range 14.50 – 41.00) was heavier than for dogs that are not English Bulldogs (13.54 kg, IQR 8.10 – 24.88, range 1.41 – 85.00) (*p* < 0.001).

**Table 1:** Descriptive statistics for demographic characteristics in English Bulldogs (*n* = 2,662) and dogs that are not English Bulldogs (*n* = 22,039) under primary veterinary care in the UK. The *P*-value represents comparison of demographic variables between English Bulldogs and dogs that are not English Bulldogs

Variable	Category	English Bulldog: count (%)	Dogs that are not English Bulldog: count (%)	*P*-value
Age (years)	< 1	425 (16.2)	2472 (11.3)	< 0.001
	1 to < 2	622 (23.7)	3218 (14.8)	
	2 to < 4	685 (26.1)	4398 (20.2)	
	4 to < 6	398 (15.2)	3404 (15.6)	
	6 to < 8	239 (9.1)	2763 (12.7)	
	≥ 8	255 (9.7)	5541 (25.4)	
Sex-neuter status	Male entire	963 (36.2)	6388 (29.1)	< 0.001
	Male neutered	379 (14.3)	5194 (23.6)	
	Female entire	956 (36.0)	5575 (25.4)	
	Female neutered	359 (13.5)	4815 (21.9)	
At/above or below mean bodyweight for breed and sex	At or above	650 (24.4)	6768 (30.7)	< 0.001
	Below	792 (29.8)	7978 (36.2)	
	Not recorded	1220 (45.8)	7293 (33.1)	
Insurance status	Insured	409 (15.4)	2942 (13.3)	0.004
	Not insured	2253 (84.6)	19,097 (86.7)	
Veterinary practice group providing clinical care	1	1166 (43.8)	9969 (45.2)	0.002
	2	503 (18.9)	3766 (17.1)	
	3	137 (5.1)	989 (4.5)	
	4	838 (31.5)	7241 (32.9)	
	5	18 (0.7)	74 (0.3)	

Of the English Bulldogs, 2,017/2,662 (75.8%) were diagnosed with ≥ 1 disorder during 2016 compared with 14,534/22,039 (65.9%) dogs that are not English Bulldogs. After using multivariable methods to account for effects of age, sex-neuter status, at/above or below mean bodyweight, insurance status and vet group, English Bulldogs had 2.04 times the odds of diagnosis with ≥ 1 disorder than dogs that are not English Bulldogs (95% confidence interval [CI] 1.85 to 2.25; *p* < 0.001).

At a specific-level of diagnostic precision, after accounting for confounding using multivariable methods, English Bulldogs had significantly increased odds of 24/43 (55.8%) specific-level disorders compared to dogs that are not English Bulldogs. These included: skin fold dermatitis (odds ratio [OR] 38.12; 95% CI 26.86 to 54.10; *p* < 0.001), prolapsed nictitating membrane gland (OR 26.79; 95% CI 18.61 to 38.58; *p* < 0.001), mandibular prognathism (OR 24.32; 95% CI 13.59 to 43.53; *p* < 0.001), BOAS (OR 19.20; 95% CI 13.31 to 27.69; *p* < 0.001) and interdigital cyst (OR 12.96; 95% CI 8.95 to 18.76; *p* < 0.001). Conversely, English Bulldogs had significantly reduced odds for 6/43 (14.0%) specific-level disorders compared to dogs that are not English Bulldogs. These included: retained deciduous tooth (OR 0.02; 95% CI 0.01 to 0.17; *p* < 0.001), lipoma (OR 0.06; 95% CI 0.01 to 0.40; *p* = 0.004), periodontal disease (OR 0.23; 95% CI 0.18 to 0.30; *p* < 0.001), pruritus (OR 0.25; 95% CI 0.13 to 0.46; *p* < 0.001) and flea infestation (OR 0.40; 95% CI 0.26 to 0.61; *p* < 0.001) (Table 2).

**Table 2:** Multivariable logistic regression odds ratios with corresponding 95% confidence intervals (CI*) for the combined list from the 30 most common disorders in English Bulldogs and in dogs that are not English Bulldogs at a *specific-level of diagnostic precision* recorded in dogs under primary veterinary care at UK practices participating in the VetCompass™ Programme from January 1^st^ 2016 to December 31^st^, 2016. Model variables accounted for included age, sex-neuter status, at/above or below mean adult bodyweight, insurance status and vet group. Specific-level precision describes the original extracted terms at the maximal diagnostic precision recorded within the clinical notes

Specific-level disorder	English Bulldog: Count (%)	Dogs that are not English Bulldog: Count (%)	Odds ratio	95% CI*	*P*-value
Skin fold dermatitis	178 (6.7)	45 (0.2)	38.12	26.86 to 54.10	< 0.001
Prolapsed nictitating membrane gland	151 (5.7)	39 (0.2)	26.79	18.61 to 38.58	< 0.001
Mandibular prognathism	55 (2.1)	15 (0.1)	24.32	13.59 to 43.53	< 0.001
Brachycephalic obstructive airway syndrome (BOAS)	112 (4.2)	43 (0.2)	19.20	13.31 to 27.69	< 0.001
Interdigital cyst	70 (2.6)	58 (0.3)	12.96	8.95 to 18.76	< 0.001
Keratoconjunctivitis sicca (KCS)	52 (2.0)	71 (0.3)	12.24	8.33 to 17.98	< 0.001
Entropion	104 (3.9)	69 (0.3)	11.61	8.44 to 15.98	< 0.001
Demodicosis	57 (2.1)	45 (0.2)	7.99	5.31 to 12.02	< 0.001
Pododermatitis	129 (4.8)	283 (1.3)	4.69	3.75 to 5.85	< 0.001
Pyoderma	112 (4.2)	317 (1.4)	3.50	2.78 to 4.40	< 0.001
Moist dermatitis	74 (2.8)	193 (0.9)	3.47	2.62 to 4.61	< 0.001
Dermatitis	53 (2.0)	156 (0.7)	3.07	2.21 to 4.25	< 0.001
Post-operative complication	56 (2.1)	146 (0.7)	2.91	2.11 to 4.01	< 0.001
Skin lesions	56 (2.1)	154 (0.7)	2.85	2.07 to 3.91	< 0.001
Otitis externa	431 (16.2)	1600 (7.3)	2.74	2.43 to 3.09	< 0.001
Cryptorchidism	51 (1.9)	121 (0.5)	2.63	1.86 to 3.71	< 0.001
Atopic dermatitis	54 (2.0)	250 (1.1)	2.24	1.65 to 3.04	< 0.001
Allergy	78 (2.9)	345 (1.6)	2.16	1.67 to 2.79	< 0.001
Conjunctivitis	112 (4.2)	486 (2.2)	1.94	1.57 to 2.41	< 0.001
Obesity	253 (9.5)	1559 (7.1)	1.79	1.54 to 2.07	< 0.001
Umbilical hernia	58 (2.2)	207 (0.9)	1.68	1.24 to 2.28	0.001
Post-operative wound	51 (1.9)	260 (1.2)	1.55	1.13 to 2.12	0.006
Patellar luxation	41 (1.5)	227 (1.0)	1.51	1.07 to 2.13	0.018
Overgrown nail(s)	187 (7.0)	1224 (5.6)	1.37	1.17 to 1.62	< 0.001
Gastroenteritis	51 (1.9)	294 (1.3)	1.34	0.98 to 1.83	0.068
Skin mass	46 (1.7)	459 (2.1)	1.33	0.97 to 1.83	0.073
Osteoarthritis	36 (1.4)	520 (2.4)	1.29	0.90 to 1.84	0.164
Kennel Cough	34 (1.3)	215 (1.0)	1.27	0.88 to 1.84	0.204
Foreign body	46 (1.7)	276 (1.3)	1.23	0.89 to 1.69	0.213
Wound	31 (1.2)	246 (1.1)	1.02	0.70 to 1.49	0.927
Anal sac impaction	115 (4.3)	1060 (4.8)	1.01	0.82 to 1.23	0.960
Skin cyst	20 (0.8)	243 (1.1)	0.94	0.59 to 1.50	0.787
Claw injury	31 (1.2)	308 (1.4)	0.88	0.59 to 1.29	0.501
Vomiting	74 (2.8)	670 (3.0)	0.86	0.67 to 1.10	0.233
Diarrhoea	94 (3.5)	843 (3.8)	0.84	0.67 to 1.05	0.133
Aggressive	44 (1.7)	498 (2.3)	0.79	0.57 to 1.09	0.145
Lameness	50 (1.9)	580 (2.6)	0.77	0.57 to 1.04	0.088
Heart murmur	17 (0.6)	473 (2.1)	0.47	0.29 to 0.76	0.002
Flea infestation	23 (0.9)	454 (2.1)	0.40	0.26 to 0.61	< 0.001
Pruritus	10 (0.4)	360 (1.6)	0.25	0.13 to 0.46	< 0.001
Periodontal disease	63 (2.4)	2786 (12.6)	0.23	0.18 to 0.30	< 0.001
Lipoma	1 (0.0)	320 (1.5)	0.06	0.01 to 0.40	0.004
Retained deciduous tooth	1 (0.0)	225 (1.0)	0.02	0.01 to 0.17	< 0.001

At a grouped-level of diagnostic precision, after accounting for confounding using multivariable methods, English Bulldogs had significantly increased odds for 17/34 (50.0%) grouped-level disorders compared to dogs that are not English Bulldogs. These included: congenital disorder (OR 7.55; 95% CI 5.29 to 10.76; *p* < 0.001), tail disorder (OR 6.01; 95% CI 3.91 to 9.24; *p* < 0.001), lower respiratory tract disorder (OR 5.50; 95% CI 4.11 to 7.35; *p* < 0.001), ophthalmological disorder (OR 4.07; 95% CI 3.63 to 4.56; *p* < 0.001) and upper respiratory tract disorder (OR 3.96; 95% CI 3.44 to 4.56; *p* < 0.001). Conversely, English Bulldogs had significantly reduced odds of 3/34 (8.8%) grouped-level disorders compared to dogs that are not English Bulldogs. These were: dental disorder (OR 0.25; 95% CI 0.20 to 0.31; *p* < 0.001), spinal cord disorder (OR 0.31; 95% CI 0.14 to 0.71; *p* = 0.005) and appetite disorder (OR 0.43; 95% CI 0.20 to 0.91; *p* = 0.028) (Table 3).

**Table 3:** Multivariable logistic regression odds ratios with corresponding 95% confidence interval (CI*) for the combined list from the 30 most common disorders in English Bulldogs and in dogs that are not English Bulldogs at a *grouped-level of diagnostic precision* recorded in dogs under primary veterinary care at UK practices participating in the VetCompass™ Programme from January 1^st^ 2016 to December 31^st^, 2016. Model variables accounted for included age, sex-neuter status, at/above or below mean bodyweight, insurance status and vet group. Grouped-level precision describes the original extracted terms mapped to a general level of diagnostic precision

Grouped-level disorder	English Bulldog: Count (%)	Dogs that are not English Bulldog: Count (%)	Odds ratio	95% CI*	*P*-value
Congenital disorder	70 (2.6)	61 (0.3)	7.55	5.29 to 10.76	< 0.001
Tail disorder	39 (1.5)	54 (0.2)	6.01	3.91 to 9.24	< 0.001
Lower respiratory tract disorder	76 (2.9)	176 (0.8)	5.50	4.11 to 7.35	< 0.001
Ophthalmological disorder	531 (19.9)	1521 (6.9)	4.07	3.63 to 4.56	< 0.001
Upper respiratory tract disorder	326 (12.2)	778 (3.5)	3.96	3.44 to 4.56	< 0.001
Urinary system disorder	86 (3.2)	267 (1.2)	3.55	2.74 to 4.60	< 0.001
Skin disorder	779 (29.3)	2750 (12.4)	3.28	2.97 to 3.61	< 0.001
Abscess	19 (0.7)	53 (0.2)	2.98	1.73 to 5.13	< 0.001
Ear disorder	456 (17.1)	1796 (8.1)	2.57	2.29 to 2.89	< 0.001
Male reproductive system disorder	68 (2.6)	196 (0.9)	2.56	1.91 to 3.42	< 0.001
Brain disorder	65 (2.4)	377 (1.7)	2.22	1.68 to 2.93	< 0.001
Complication associated with clinical care	105 (3.9)	412 (1.9)	2.02	1.61 to 2.52	< 0.001
Female reproductive disorder	94 (3.5)	324 (1.5)	2.01	1.58 to 2.57	< 0.001
Collapsed	29 (1.1)	270 (1.2)	1.60	1.08 to 2.39	0.020
Hernia	62 (2.3)	254 (1.1)	1.53	1.15 to 2.04	0.004
Musculoskeletal disorder	260 (9.8)	1921 (8.7)	1.52	1.32 to 1.75	< 0.001
Adverse reaction to drug	31 (1.2)	165 (0.7)	1.39	0.94 to 2.07	0.100
Claw/nail disorder	224 (8.4)	1566 (7.1)	1.31	1.13 to 1.52	< 0.001
Foreign body	46 (1.7)	279 (1.3)	1.21	0.88 to 1.67	0.247
Neoplasia	101 (3.8)	1220 (5.5)	1.13	0.91 to 1.40	0.281
Traumatic injury	113 (4.2)	815 (3.7)	1.07	0.87 to 1.31	0.536
Enteropathy	306 (11.5)	2326 (10.5)	1.04	0.91 to 1.19	0.544
Thin	36 (1.4)	332 (1.5)	1.03	0.72 to 1.46	0.881
Anal sac disorder	132 (5.0)	1236 (5.6)	1.01	0.83 to 1.21	0.951
Parasite infestation	113 (4.2)	842 (3.8)	0.99	0.81 to 1.22	0.949
Mass	87 (3.3)	1186 (5.4)	0.99	0.79 to 1.25	0.937
Undesirable behaviour	124 (4.7)	1169 (5.3)	0.94	0.77 to 1.13	0.498
Heart disease	45 (1.7)	670 (3.0)	0.88	0.64 to 1.20	0.417
Endocrine system disorder	9 (0.3)	201 (0.9)	0.75	0.38 to 1.47	0.396
Lethargy	22 (0.8)	280 (1.3)	0.73	0.47 to 1.14	0.171
Incontinence	8 (0.3)	206 (0.9)	0.59	0.29 to 1.22	0.156
Appetite disorder	7 (0.3)	180 (0.8)	0.43	0.20 to 0.91	0.028
Spinal cord disorder	6 (0.2)	232 (1.0)	0.31	0.14 to 0.71	0.005
Dental disorder	82 (3.1)	3140 (14.2)	0.25	0.20 to 0.31	< 0.001

## Discussion

Recent research has sought to explain why purchasers remain undeterred by the negative implications of brachycephalic conformation for animal health and welfare while instead prioritizing their characteristic appearance, personality and perceived suitability for certain lifestyles, often based on their low exercise requirements/ability [41–43]. With over 90% of current English Bulldog owners stating they would re-purchase this breed again [44], the current popularity of the English Bulldog shows little signs of abating. Consequently, understanding the development of conformation-related disease within the breed, and accurately documenting the disease burden and predispositions within the current breed population [6] are key to assessing and, if possible, redressing some of the main health issues in English Bulldogs. In line with the study hypothesis, the four predispositions with the highest odds in English Bulldogs are all directly associated with the extreme conformation that defines the English Bulldog breed: skin fold dermatitis (× 38.12) [8], prolapsed nictitating membrane gland (× 26.69) [45], mandibular prognathism (× 24.32) [8] and BOAS (19.20) [14]. This evidence supports calls for urgent action to redefine the English Bulldog away from its current extreme conformation and instead to move the breed rapidly towards a moderate conformation on welfare grounds. The results of the current study support the study hypothesis that English Bulldogs show more predispositions than protections among common disorders overall. In line with prior evidence of frequent health issues in the breed [10], these current results support the previous assessments of poor health in (English) Bulldog populations dating back over a hundred years. In 1901, seven years was considered ‘quite an old age’ for a Bulldog; in 1954, critics debated why the breed had ‘a shorter expectation of life’ than others [46, 47]. More recently, Kennel Club surveys of English Bulldog mortality in 2004 and 2014 respectively reported median and mean ages of death of just over six years [48, 49]. Analysis of mortality data from primary-care veterinary clinical records reported a median longevity of 7.2 years for English Bulldogs in the UK [50]. Supporting a shorter lifespan overall in English Bulldogs, the current study reports the median age of English Bulldogs surveyed in 2016 (2.65 years) as significantly younger than of surveyed dogs that are not English Bulldogs (4.42 years). This could be partly explained by a population that skews towards young animals because of a growing popularity of the breed, as seen in steadily increasing Kennel Club registration figures over the last decade [21] and reported in primary-care veterinary practice [50]. However, given that only 9.7% of English Bulldogs in this study were aged over eight years compared with 25.4% of the dogs that are not English Bulldogs, this suggests that relatively few English Bulldogs reach the advanced ages that are typical of other breeds, in line with previous reports, and supports a view of poor overall health in the breed [10, 21].

Likewise, the disease predispositions reported in the current study for English Bulldogs show striking parallels to those previously attributed to the breed. For example, the disorder with the highest predisposition in the current study of English Bulldogs in 2016 was skin fold dermatitis, which was also the most frequently reported disease for Bulldogs in the 1962 BSAVA survey [20]. Similarly, the predisposition of the English Bulldog to prolapsed nictitating membrane gland and to entropion was recognised by veterinary ophthalmologists in 1914 [51]; these conditions were respectively the second and seventh highest disease predispositions in the current study of English Bulldogs in 2016. Mandibular prognathism was the third highest predisposition of English Bulldogs in 2016. This is unsurprising, since this attribute has been a deliberate feature of the Bulldog breed standard since the nineteenth century, with the original wording, that ‘the lower jaw should project considerably in front of the upper, and turn up’, recently modified to require a ‘slightly projecting’ lower jaw instead [8, 52]. The fourth highest predisposition for English Bulldogs in 2016 was BOAS, again reiterating the long-documented propensity of the breed for upper respiratory disorders, reported since the late nineteenth century and previously quantified in 1962 with a high reported incidence of ‘elongated soft palate’ [3, 20]. Thus, the leading predispositions for disease in English Bulldogs, as determined by the current analysis of 2016 data, broadly correspond to disorders that have been long associated with the breed. This provides strong evidence supporting the validity of previous qualitative assessments of breed-related disease in English Bulldogs, but also revives the perennial question of why, since these diseases have been repeatedly documented as impairing Bulldog health for over a century, the breed nevertheless remains so commonly affected by these problems.

In assessing which predispositions and protections to disease particularly differentiate English Bulldogs from the remaining general canine population, it is helpful to focus particularly on ultra-predispositions by breed: i.e. those conditions which are seen at particularly high levels in English Bulldogs, with odds more than four times higher than in dogs that are not English Bulldogs [53]. At the specific disorder level of analysis, the English Bulldog population in the current study showed ultra-predispositions to nine recorded disorders. Of these, four concerned diseases of the skin (skin fold dermatitis, interdigital cyst, demodicosis and pododermatitis). Moreover, additional skin diseases also heavily predominated among the specific-level disorders that were seen between two and four times more frequently in English Bulldogs than other dogs; these comprised pyoderma, moist dermatitis, dermatitis, skin lesions, atopic dermatitis and the non-specific descriptor of ‘allergy’, and also included otitis externa, which is often clinically linked to other allergic skin disease [54]. These various dermatological conditions were necessarily differentiated by the different descriptors used by the originating primary care clinicians. When processing the data, each disorder was recorded once to the greatest possible level of clinical precision. However, these descriptors will inevitably potentially refer to similar or overlapping pathologies, and the certainty of the clinical diagnoses will not necessarily have been equally rigorous or precise in all cases. Therefore, it may be more justified to consider these linked and overlapping conditions at the grouped-level of skin disorders, where 29.3% of Bulldogs were reported with skin disease compared to 12.4% of the general canine population; with an odds ratio of 3.28 after accounting for confounding. These findings confirm that English Bulldogs carry substantially increased risk of skin disease compared with other dogs. While not all skin disease is directly related to exaggerated conformation, skin fold dermatitis, which by definition only occurs in folded skin, was the specific-level disorder to which English Bulldogs were most predisposed and showed a dramatically increased odds ratio of 38.12, presumably reflecting a dangerous synergy of a wider propensity to skin disease combined with the particular issue of wrinkled facial and tail-base skin caused by the brachycephalic and tail conformations that define the English Bulldog breed [8]. Although the English Bulldog’s underlying propensity for skin disease is likely underpinned by complex polygenic and environmental factors, selection away from skin folds represents a comparatively simple biological challenge that should dramatically reduce the prevalence of skin fold dermatitis; however, given the longstanding desire by humans for this feature in English Bulldogs, achieving this welfare-based modification may represent a far greater challenge for human behaviour change than for breeding biology.

Three ophthalmic conditions featured among the nine ultra-predispositions in English Bulldogs at the specific level of disorder: prolapsed nictitating membrane gland, keratoconjunctivitis sicca and entropion. A predisposition of English Bulldogs to entropion (in-turned eyelids) has been reported for over a hundred years [51]. This finding was confirmed by the current study, where 3.9% of the English Bulldogs surveyed were diagnosed with entropion, and the breed showed a markedly increased odds ratio of 11.61 compared to dogs that are not English Bulldogs. Entropion, like skin fold dermatitis, is commonly seen in brachycephalic breeds and is generally attributed to the excess facial skin that results from a foreshortened facial structure; hence, it is usually considered a conformation related disease [55, 56].

Both prolapsed nictitating membrane gland and keratoconjunctivitis sicca were also reported in the current study as ultra-predispositions in English Bulldogs. A diagnosis of prolapsed nictitating membrane gland was 26.79 times more likely in English Bulldogs than in dogs that are not English Bulldogs, while keratoconjunctivitis sicca showed a less extreme but still markedly high odds ratio of 12.24. Yet, despite the greatly elevated odds ratio of prolapsed nictitating membrane gland in English Bulldogs, the 5.7% of English Bulldogs in the wider general population of dogs diagnosed with prolapsed nictitating membrane gland in this study comprise a much lower incidence than the 75% reported in previous Kennel Club surveys of pedigree English Bulldogs, as discussed above [25]. This may indicate genuine and/or methodological differences between the surveyed populations. It may also reflect under-recording of prolapsed nictitating membrane gland in primary care clinical records. The condition (known colloquially as ‘cherry eye’) often manifests in young dogs and is well known within the English Bulldog breed community, where both surgical excision of the gland (often under local anaesthesia) and surgical replacement (recommended by ophthalmologists as a superior technique) are considered possible treatments [57]. Consequently, dogs might be treated for this condition before sale or without the knowledge of primary care practices, potentially resulting in under-recording or under-diagnosis of this disorder in this study. Moreover, although several aetiopathological pathways are proposed for keratoconjunctivitis sicca, one possible cause is following the surgical treatment of a prolapsed nictitating membrane gland, particularly if the tissue is excised rather than replaced (because the nictitating membrane gland contains secretory cells that contribute to lacrimal production) [58, 59]. Therefore, the ultra-predisposition to keratoconjunctivitis sicca noted in the current study may also reflect previous sub-optimal surgical treatment of prolapsed nictitating membrane gland. Thus, the ultra-predisposition of English Bulldogs to these two disorders in this study may be causally linked; further investigation of this association would inform future recommendations for the treatment of prolapsed nictitating membrane gland.

The predisposition of English Bulldogs to BOAS is well documented [14, 60], and is of sufficient clinical concern that The Kennel Club and the University of Cambridge have jointly launched a Respiratory Function Grading Scheme to facilitate selective breeding away from this condition in English Bulldogs and other brachycephalic breeds [61]. It has previously been suggested that up to 45% of English Bulldogs may exhibit clinical signs of BOAS [62]. In the current study, despite a striking odds ratio of 19.2 for BOAS in English Bulldogs compared with dogs that are not English Bulldogs, only 4.2% of the Bulldogs surveyed were formally diagnosed with BOAS during 2016, which is a surprisingly small proportion when compared with previous studies. While it is possible that this reflects biological difference between surveyed populations in different studies, it is much more likely that this discrepancy is chiefly due to normalisation of the condition in primary care practice [63]. For disorders that are so frequent that these become almost a typical expectation for certain breeds, then owners and even veterinarians may no longer even consider these conditions as disorders but instead may normalise these ‘just being part of the breed’ [42, 64, 65]. Normalisation of English Bulldogs with clinical signs of BOAS such as stertor, stridor or stenotic nares as ‘not unhealthy’ appears to be common in the UK, with the owners of over half of brachycephalic dogs with BOAS perceiving these clinical signs (e.g. increased and abnormal respiratory noise) as ‘normal for the breed’ in two separate populations [63, 66].

Mandibular prognathism constitutes a different type of ultra-predisposition in the English Bulldog population surveyed and raises the thorny question of why a conformation that would be considered a serious health issue in one dog breed can be actively selected as a desirable trait in another. As previously mentioned, ‘undershot’ jaw – the physical appearance described by the term mandibular prognathism – has been an explicit requirement of the Bulldog breed standard for over a century [8]. Although mandibular prognathism was the third highest predisposition recorded in the current study, with 24.32 times increased odds in English Bulldogs, this still meant that only 2.1% of the overall English Bulldog population were reported with this condition. Given that mandibular prognathism is a requirement of the breed standard, it is probable that most, if not all, English Bulldogs show this disorder and therefore the current results represent a vast understatement of the real situation of a condition that is ‘normal for the breed’ regardless of not being ‘normal for a dog’ and which therefore will generally go unrecorded in the clinical notes. Similarly, at the grouped level of disorder, ‘tail disorders’ constituted an ultra-predisposition in English Bulldogs with an odds ratio of 6.01 that were recorded in 1.5% of the population. However, since modern English Bulldogs are virtually homozygous for the genetic mutation that causes the ‘screw’ tail, it is probable that many English Bulldogs in this survey similarly went unrecorded for tail abnormalities because these disorders were considered ‘normal for the breed’ [67]. These findings suggest that veterinarians are also becoming normalised to disorders that are accepted as ‘normal for breed’ that would be considered as severe and noteworthy in most other breeds, or alternatively that many veterinarians eventually reach a state of learned helplessness after prolonged contact with events that are outside their personal control [68].

Given these common cognitive biases in owners and veterinarians towards accepting conditions as ‘normal’ in English Bulldogs that would otherwise be considered as disorders in breeds with less extreme conformations, it is therefore all the more striking that English Bulldogs are the breed with the highest proportions of predispositions among common disorders from the breeds that have been reported to date using this VetCompass methodology. English Bulldogs showed predispositions for 55.8% (24/43) of specific-level disorders. In comparison, French Bulldogs showed predispositions for 46.5% (20/43) of disorders [53], Labrador Retrievers showed 34.3% (12/35) predispositions [69] and Staffordshire Bull Terriers showed 11.1% (4/36) predispositions [70]. These results provide strong evidence supporting substantially poorer overall health in English Bulldogs and French Bulldogs compared to Labrador Retrievers and Staffordshire Bull Terriers.

Although dogs had long been divided into loosely-recognised types, the concept of clearly differentiated and strictly delineated breeds was an invention of the Victorian era [2]. Traditional breeds were defined, and new ones created, through detailed descriptions of their physical attributes in ‘breed standards’. Since the visible differences between breeds were key determinants of this new breed system, this evolving culture catalysed and accelerated the exaggeration of distinctive features in some pre-existing types of dogs, both through deliberate efforts to secure their recognition as distinct ‘breeds’ and as a natural consequence of the financial and competitive rewards that often followed the production of dogs with more extreme conformation for the show-ring [3]. In consequence, the modern breeds that we know today can be ranked along a spectrum from mild to extreme conformational exaggeration, ranging from canine-typical (mild conformational exaggeration) to canine-divergent (severe conformational exaggeration) [13, 14]. The combined proportion of predispositions and protections to disease within a breed could be used as a measure of overall health divergence between that breed and the mainstream canine population. Using this measure, English Bulldogs showed health divergence from other dogs for 69.8% (30/43) of specific-level disorders. French Bulldogs showed a similarly high level of health divergence, differing in 77.1% (31/43) from all remaining dogs [53]. In comparison, Labrador Retrievers differed from other dogs for 54.3% (19/35) of disorders [69] while Staffordshire Bull Terriers showed a health divergence metric of just 25.0% (9/36) from other dogs [70]. The two Bulldog breeds with extreme brachycephaly thus both showed notably higher predispositions to disease as well as more disease divergence from other dogs than either Labrador Retrievers or Staffordshire Bull Terriers, although predispositions were relatively higher among English Bulldogs and protections among French Bulldogs. Moreover, as discussed earlier, English Bulldogs showed multiple ultra-predispositions to disease, with odds more than four times higher than dogs that are not English Bulldogs in 9/43 (20.9%) of recorded specific level disorders, while French Bulldogs showed ultra-predispositions to disease in 11/43 (25.6%) of specific-level disorders [53]. In contrast, neither Labrador Retrievers nor Staffordshire Bull Terriers showed any ultra-predispositions to disease at all, with their highest predispositions being an odds ratio of 2.83 for osteoarthritis in Labrador Retrievers and of 2.06 for seizure disorder in Staffordshire Bull Terriers [69, 70]. Overall, therefore, these measures of disease predispositions, divergences and ultra-predispositions seen in English Bulldogs reveal a worrying story of a breed with more disease predispositions than other dogs, which differs widely from other dogs in their patterns of disease and is characterised by several ultra-predispositions to disease. With many of these ultra-predispositions to disease in the English Bulldog being linked to their characteristic extreme physical features, these results broadly confirm an unchanging link between exaggerated conformation and disease first flagged as a concern over a century ago [3].

All breeds, by definition, are different in some ways from the average for the canine population and therefore are likely to show some breed predispositions to, and protections from, disease [6, 8]. For English Bulldogs, the most marked protections for disease, where dogs were less than half as likely to show the specific level disorder as other dogs (an OR of < 0.5), were heart murmur (OR 0.47), flea infestation (OR 0.40), pruritus (OR 0.25), periodontal disease (OR 0.23), lipoma (OR 0.06) and retained deciduous tooth (OR 0.02). At the grouped disorder level, marked protections (OR < 0.5) were reported for appetite disorder (OR 0.43), spinal cord disorder (0.31) and dental disorder (0.25). Some of these protections are somewhat surprising; for example, given the breed’s documented high incidence of hemivertebrae and other vertebral malformations [15, 71–73], it is unexpected but reassuring to find that this does not apparently translate into a high level of spinal cord disorder, and may be explained by previous findings that hemivertebrae is more commonly associated with a neurologically normal phenotype in English Bulldogs than for Pugs (Ryan et al., 2017). It is also surprising that a breed with a high prevalence of many other descriptors for skin disease has an apparent protection for pruritus: perhaps this descriptor was subsumed within other more clinically precise descriptors, such as dermatitis or atopic dermatitis, and hence appears artefactually low. It may be that the recorded protection for periodontal disease and dental disorder is linked to the normalisation phenomena described above, but this conclusion is speculative. Other apparent protections for heart murmur and lipoma may indicate true disease protections in the breed, which could be explored in future studies.

Over recent years, The Kennel Club has made concerted efforts to alleviate drivers for extreme conformation from the show-ring. Since 2012, The Kennel Club has identified the English Bulldog as a breed at particularly high risk of conformation-related disease: the breed is currently grouped in Category 3 on The Kennel Club’s Breed Watch list, with show judges urged to prioritise health in their show-ring decisions [74]. Moreover, The Kennel Club breed standard for the English Bulldog has undergone iterative revisions over recent decades, intended to modify its wording to encourage the selection of dogs towards less extreme conformation [8]. Given that only a third of UK dogs are estimated to be registered with The Kennel Club [21]; and that even among this registered subset only a very small proportion are specifically bred for the show-ring [75], the impact of these measures on the general population of English Bulldogs is likely to be minimal. Although show-ring practices have undoubtedly had a profound historical influence in determining the shape of the modern English Bulldog [3], and most dogs described as English Bulldogs today will ultimately be descended from dogs bred for the show-ring in the past, it is easy to overstate the current significance of the show world and Kennel Club breed standard among English Bulldog breeders. While breed standard modifications are to be welcomed, they directly drive change only in the show population, and even then only if judges abide by them [76]. The show Bulldog community argues that its enthusiasts now discourage ultra-extreme conformation and that judges are instructed not to award prizes to dogs with obvious physical compromise. Conversely, the show community claims that ‘overdone’ dogs with extreme conformation tend to be bred outwith the show community by breeders who also select for non-standard ‘novel’ colours, such as merle, which typically command higher prices (Bulldog Breed Council, 2021a). Further research is needed to investigate the social factors and local subcultures that drive and differentiate these breeding priorities, and how best to encourage behavioural change in each group.

The current study had some limitations in addition to those previously reported for the application of primary care veterinary data for canine research [39, 77]. This study relied on the breed identifications recorded on veterinary practice databases. The English Bulldogs in the current study included dogs registered with The Kennel Club as well those that are not. The study dogs are therefore likely to show a range of conformations, ranging from ultra-extreme to relatively moderate, that are consistent with our current acceptance of what constitutes an English Bulldog. Disorder risk for English Bulldogs was compared against all remaining dogs that were not English Bulldogs. Given that 18.74% of dogs in the UK are brachycephalic, this means that a large proportion of the comparator group of dogs in the current study were also brachycephalic. This may have led to a masking effect for disorders linked to the brachycephalic conformation in the current study and suggests that the true levels of predisposition to disorders linked to brachycephaly in the English Bulldog could be much higher than reported here.

Future work could include prospective cohort studies that compare disorder predisposition between English Bulldogs with moderate conformation and English Bulldogs with extreme conformation to evaluate potential welfare gains from conformational change within the breed. Repeating the current study design at defined intervals could also monitor real-world changes in the health of the English Bulldog over time following efforts by UK national groups such as the Brachycephalic Working Group [27].

## Conclusions

In providing further evidence that English Bulldogs experience unusually high levels of disease and show multiple predispositions and ultra-predispositions to disease, much of which is intrinsically related to their conformation, this study broadly confirms long-standing assertions that the health and welfare of English Bulldog is heavily compromised. Yet, despite extensive evidence and wide dissemination of the health issues and their serious negative welfare impacts for these dogs, it seems that many prospective owners are still not discouraged from English Bulldog ownership [41, 42]. Even after many years of campaigning to increase public awareness of brachycephalic health issues and a public message from the UK Brachycephalic Working Group to ‘stop and think before buying a flat-faced dog’ [27], English Bulldogs remain extremely popular, with their current rank as the fourth highest in Kennel Club registrations in 2020 reprising a peak in popularity from over a century ago [21, 27]. While the current study cannot expect to solve the conundrum of ownership and health issues in the English Bulldog, it does provide a disorder benchmark with some novel health metrics that could be used to explore and promote changes in population health for English Bulldogs over time in the future and be used as an evidence base to challenge the ethical and welfare acceptability of perpetuating the current extreme conformation of some breeds with known high disease burdens. Immediate redefinition of the English Bulldog towards a moderate conformation is strongly advocated to avoid the UK joining the growing list of countries where breeding of English Bulldogs is banned.

## Data Availability

The datasets generated during and/or analysed during the current study will be made available at the RVC Research Online repository.

## Abbreviations

BHCP: Breed Health and Conservation Plan
BOAS: Brachycephalic obstructive airway syndrome
BSAVA: British Small Animal Veterinary Association
CI: Confidence interval
EPR: Electronic patient record
IQR: Interquartile range
OR: Odds ratio
